# Exploring bubble oscillation and mass transfer enhancement in acoustic-assisted liquid-liquid extraction with a microfluidic device

**DOI:** 10.1038/srep12572

**Published:** 2015-07-30

**Authors:** Yuliang Xie, Chandraprakash Chindam, Nitesh Nama, Shikuan Yang, Mengqian Lu, Yanhui Zhao, John D. Mai, Francesco Costanzo, Tony Jun Huang

**Affiliations:** 1 Department of Chemical Engineering, The Pennsylvania State University, University Park, Pennsylvania 16802, USA; 2 Department of Engineering Science and Mechanics, The Pennsylvania State University, University Park, PA 16802, USA; 3 Department of Mechanical and Biomedical Engineering, City University of Hong Kong, Tat Chee Avenue, Kowloon, Hong Kong SAR; 4 Center for Neural Engineering, The Pennsylvania State University, University Park, PA 16802, USA; 5 Department of Biomedical Engineering, The Pennsylvania State University, University Park, PA 16802, USA

## Abstract

We investigated bubble oscillation and its induced enhancement of mass transfer in a liquid-liquid extraction process with an acoustically-driven, bubble-based microfluidic device. The oscillation of individually trapped bubbles, of known sizes, in microchannels was studied at both a fixed frequency, and over a range of frequencies. Resonant frequencies were analytically identified and were found to be in agreement with the experimental observations. The acoustic streaming induced by the bubble oscillation was identified as the cause of this enhanced extraction. Experiments extracting Rhodanmine B from an aqueous phase (DI water) to an organic phase (1-octanol) were performed to determine the relationship between extraction efficiency and applied acoustic power. The enhanced efficiency in mass transport via these acoustic-energy-assisted processes was confirmed by comparisons against a pure diffusion-based process.

“Acoustic-wave-assisted liquid-liquid extraction” (ALE) is a widely used procedure where target analytes are transferred and enriched across a liquid-liquid interface^1^,^2^,^3^,^4^,^5^. By introducing acoustic waves^6^,^7^,^8^,^9^,^10^,^11^,^12^,^13^,^14^,^15^, the rate of mass transfer can be increased while significantly reducing the extraction time, decreasing undesirable solvent loss, and maintaining the activity of biomolecules^16^,^17^,^18^,^19^. The superior features of ALE are a result of microcavitations, *i.e*., micro bubbles formed by interactions with acoustic waves in the liquid environment. The creation, expansion, and collapse^20^,^21^,^22^ of these microcavities^23^,^24^,^25^ facilitates molecular transfer across the liquid-liquid interface^26^. Due to its excellent performance and simple experimental setup, ALE is especially suitable for the enrichment of biomolecules^27^. For example, ALE has been used to extract biomolecules (*e.g*., DNA) from a lysed cell^29^ before analysis to enhance detection performance^30^,^31^. In a biomedical context, cell sonoporation^32^,^33^, a particular type of ALE that occurs across cell membranes, is widely used to transfer therapeutic agents and genes into cells.

Despite its wide utility, the oscillation of the cavities and the resulting induced mass transfer across the liquid-liquid interface near a single cavity is still unexplored. This is likely due to the randomness in the bubble movements and the subsequent locations of the cavitations in conventional ALE operations. Investigating this process requires the integration of multiphase fluids and the precise generation of microcavities in one device. In this research, we utilize an acoustically-driven, bubble-based microfluidic device to quantitatively investigate bubble oscillation and the induced mass transfer enhancement across a liquid-liquid interface. The problem of the random locations and sizes of microcavities is solved by trapping isolated air bubbles^34^,^35^,^36^,^37^,^38^,^39^ and by forming liquid-liquid interfaces over predefined microstructures. In addition, the oscillation of the bubble due to acoustic waves^40^,^41^,^42^,^43^ is known to facilitate molecular transport across the interface. Thus, this configuration, with the capability to control the sizes of bubbles and low-energy bubble-actuation, allows us to integrate ALE onto a microfluidic device; henceforth referred to as “on-chip ALE”. Furthermore, with this on-chip ALE using a trapped bubble, it becomes possible to optimize the operating conditions, aided by a theoretical study of non-spherical bubble behavior in the presence of acoustic waves in microsystems.

This article is organized in the following manner: First, the design of a bubble-based microfluidic device, with a hemispherical bubble and multiphase liquid, is described. Next, the oscillations of a bubble and the liquid-liquid interface are quantitatively characterized at both a single frequency and over a frequency range. Following this, a theoretical explanation for the behavior of an acoustically-driven, non-spherical trapped bubble is provided. Then the theoretically estimated and experimentally measured resonant frequencies are compared. Finally, the mass transfer enhancement is evaluated via extraction of Rhodanmine B from water to 1-octanol.

## Methods

### Experimental setup

**Figure 1:**
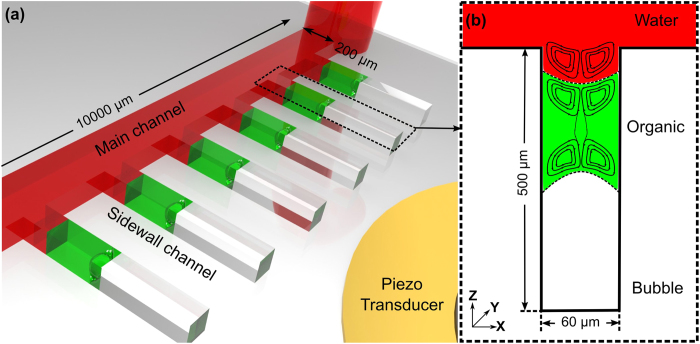
Schematic of the (**a**) experimental setup and (**b**) bubble oscillation-induced streaming near the organic-gas surface and near the water-organic solvent interface.

A polydimethylsiloxane (PDMS)-based microfluidic device was fabricated using standard soft lithography and mold replica techniques^44^. The microfluidic device comprises a main channel 200 μm × 10,000 μm × 55 μm, with 5 side channels that form T-intersections with the main channel (Fig. 1a). The intersecting side channels (Fig. 1b) have the following dimensions: 60 μm (width) × 500 μm (length) × 55 μm (depth). The patterned PDMS device was bonded onto a glass slide with oxygen plasma to seal the microfluidic channels. A piezoelectric transducer (No. 273-073, RadioShack, USA), which converted electrical inputs into acoustic waves, was bonded to the same glass slide. This piezo transducer was driven by a function generator (8116A, Hewlett Packard, USA) in either a single fixed frequency mode or in a continuous frequency sweeping mode.

### Formation of the bubble and the liquid-liquid interface

**Figure 2:**
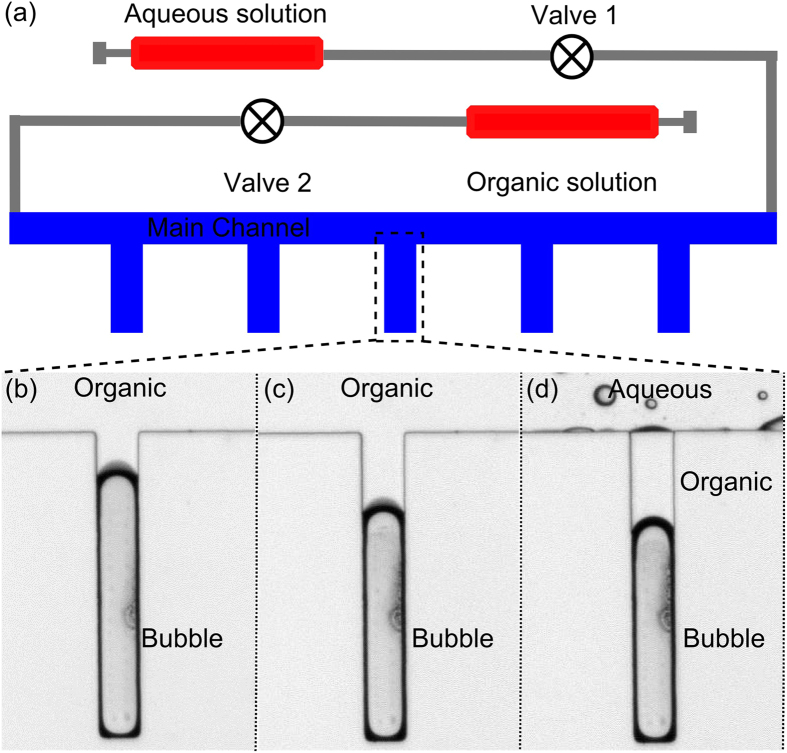
(**a**) Schematic of the experimental setup to trap bubbles and to form liquid-liquid interfaces inside the T-intersection side cavities; (**b**–**d**) Sequential flow injection forms an interface between the organic and aqueous phases on top of a bubble.

**Figure 3:**
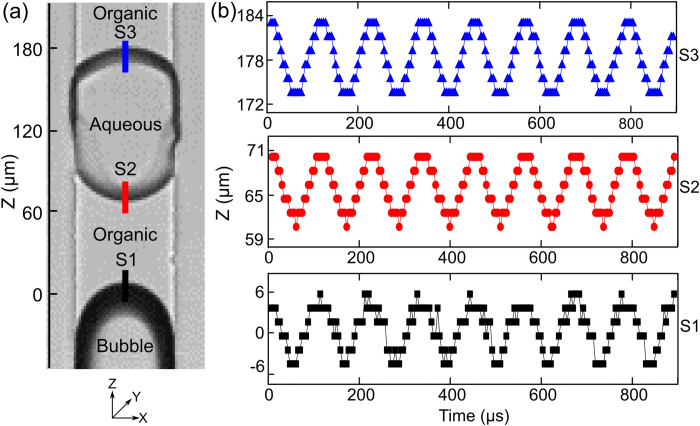
(**a**) Image showing the oscillation of the bubble and the liquid-liquid interface. The position changes in the organic-aqueous (*S*_*3*_), the aqueous-organic (*S*_*2*_), and the organic-air (*S*_*1*_) interfaces, is indicated by the blue, red, and black lines, respectively.; (**b**) The changes in positions of *S*_*1*_*, S*_*2*_, and *S*_*3*_ for an acoustic wave frequency of 10 kHz.

**Figure 4:**
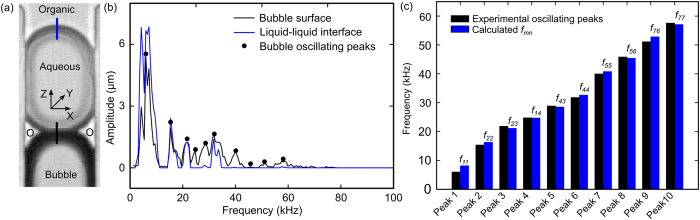
(**a**) Oscillation of the bubble and the liquid-liquid interfaces. The amplitudes of oscillation of the bubble (*S*_*1*_) and liquid-liquid interface (*S*_*3*_), are measured along the black line and blue line respectively. (**b**) Amplitude of oscillation for *S*_*3*_ and *S*_*1*_ over a frequency range of 1-100 kHz. The black dots indicate the local amplitude peaks for the bubble. (**b**) Comparison between the experimentally measured frequencies and the calculated resonant frequencies.

The air bubble (hereafter just referred to as a bubble) was trapped and the liquid-liquid interface above the bubble was formed by sequentially injecting organic and aqueous solutions into the main channel. In the experiments, two syringe pumps (neMESYS, Cetoni GmbH, Germany) were used for liquid injections. Each pump was connected to one end of the channel via tubing. A valve was installed on each tube, between the respective syringe pump and the main microchannel, to regulate flow (Fig. 2a). The simplest organic-aqueous liquid interface was formed in three steps (Fig. 2b–d). After opening both valves, an organic solution (1-octanol) was injected via a syringe pump into the main channel. An air bubble was trapped inside the side channel (Fig. 2b). Due to surface tension forces the organic liquid-air interface assumes a hemispherical shape. Next, valve 1 (Fig. 2a) was closed to stop the outflow of organic solution, while the injection of the organic solution continued to increase the pressure inside the microfluidic channel. This compressed the bubble and forced a small amount of organic liquid into the side channel (Fig. 2c). Next, injection of the organic solution was stopped and both valves were opened. Then water was injected into the main channel (Fig. 2d). Creating and organic aqueous interface. More complex combinations of interfaces (*e.g.,* interfaces shown in Figs 3a and 4a) could also be formed by changing the liquid injection sequence.

### Evaluation of mass transfer

Mass transfer across the liquid-liquid interface was investigated quantitatively by extracting Rhodanmine B from water to 1-octanol at room temperature. All chemicals were purchased from Sigma-Aldrich at analytical grade purity. Before the experiment, the Rhodanmine B was diluted in deionized (DI) water to a concentration of 10 μM from an initial stock concentration of 1 mM at 4 °C. The microfluidic system, including water-1-octanol (aqueous-organic) interface and 1-octanol-gas bubble, was driven by acoustic wave at 10 kHz, at three different driving voltages: 0, 10, or 20 V peak-to-peak (V_pp_). The extent of Rhodanmine B extraction was estimated by measuring changes in the Rhodanmine B concentration in 1-octanol every 5 s.

A CCD camera (Coolsnap HQ2, Photometrics, USA) was used to take bright-field images and fluorescent images. In the experiments with Rhodanmine B extraction, bright-field images of the side channel were recorded every 5 s. The grey-scale values from images of the liquids within the cavities were analyzed using *ImageJ* software to estimate the Rhodanmine B concentrations. The acoustic streaming (Fig. 1b) induced by bubble oscillations was visualized by the trajectories of fluorescent microparticles. The time series fluorescent images were stacked by *ImageJ* software to visualize the streaming pattern. A high-speed camera (SA4 Fastcam, Photron, USA) was used to record the oscillations of the bubble and the liquid-liquid interfaces. The camera was positioned facing the *xz* plane to record videos of bubble oscillation at 225,000 frames per second. The high-speed camera had a spatial resolution of 1 μm and a time resolution of 4 μs. As a comparison, the bubble diameter was 60 μm; the bubble was actuated at 10–100 kHz, which has a period from 10-100 μs. Thus our imaging system could capture the oscillations without aliasing effects. Based on the captured videos, the oscillations of the liquid-liquid interface and bubble were analyzed using a MATLAB program, which analyzed the grey value intensity along the center-line of the side-wall cavity in each frame, and considered the darkest points as the positions of the interfaces. The amplitudes of each interface are calculated using the displacements of the interfaces over several cycles.

## Results and Discussion

### Single-frequency oscillations

The oscillation behavior of the liquid-liquid interface was first tested at a single set frequency of 10 kHz at a driving voltage 12 V_pp_. To analyze the oscillation of the bubble, we will now focus the discussion only on the interface configuration shown in Fig. 3a; the other interface configurations which are simplified versions of this interface with similar oscillatory behaviors are discussed in the Supplementary Information (Fig. S1). The changes in the positions of the bubble and the liquid-liquid interface during bubble oscillations are displayed in Fig. 3b. The bubble surface and the interface appear darker in the picture due to the light refraction at the interfaces. The motion of the middle points for the organic-air bubble interface (*S*_*1*_), the aqueous-organic interface (*S*_*2*_), and the organic-aqueous interface (*S*_*3*_) were measured along the z direction and are indicated by the black, red, and blue curves, respectively in Fig. 3b. The maximum amplitude of bubble oscillation was found to be approximately 6 μm. It was found that no phase-lag exists in the oscillations among *S*_*1*_, *S*_*2*_, and *S*_*3*_. The absence of phase lag can be attributed to the fact that the wavelengths (15–150 cm) at our operating frequencies (10–100 kHz) are much larger than the characteristic dimension of our device (60 μm). So, the system is considered as a lumped model at these operating frequencies.

Since liquids are less compressible than gases^45^, the liquids used in the side cavity in our experiments (*i.e*., water and 1-octanol) are assumed to be incompressible compared to the air bubbles. Thus, the oscillations of both *S*_*2*_ and *S*_*3*_, which determine the mass transfer, can be characterized by determining the oscillatory properties of *S*_*1*_ alone. However, the amplitudes of the oscillation of *S*_*1*_, *S*_*2*_, and *S*_*3*_ were not exactly the same. This could be due to differences in the curvatures resulting from different contact angles for the organic-gas and aqueous-organic interfaces. Also, the slight differences observed in the amplitudes of the interfaces might be due to the experimental error in capturing the maximum displacement amplitude (owing to the finite frame capture rate).

### Oscillations at multiple frequencies

Since the bubble oscillation is the driving factor for the extraction, the microsystem is studied over a range of frequencies to identify the resonant frequencies. The input frequency of the electrical signal to the piezo transducer was swept linearly from 1 kHz to 100 kHz over a 300 ms period, at a voltage 12 V_pp_. First, the frequency response of the piezo transducer was measured in order to exclude the effects of the transducer resonances. These measurements are provided in the Supplementary Information. The piezo transducer has several resonant peaks located between 5 and 6 kHz, 30 and 35 kHz, at 80 kHz, and near 100 kHz (Fig. S2, Supplementary Information). The same piezo transducer is then used to activate the bubble and the liquid-liquid interface system.

Using the liquid-liquid interface configuration shown in Fig. 4a, the oscillation amplitudes of the organic-aqueous interface (*S*_*3*_, blue line in Fig. 4a) and the organic-air interface (*S*_*1*_, black line in Fig. 4a) were recorded and analyzed. Several distinctive features were revealed from the amplitude trends of the *S*_*1*_ and *S*_*3*_ oscillations over the range of the frequencies: (1) the oscillation of *S*_*3*_ was similar to *S*_*1*_, confirming the dependence of the liquid-liquid interface behavior on the bubble motion. (2) The oscillation amplitudes for both *S*_*1*_ and *S*_*3*_ tend to decrease as the frequency increased, independent of the input voltage. (3) The oscillation amplitudes of *S*_*1*_ and *S*_*3*_ had several maximums (black dots in Fig. 4b). These peaks were at frequencies that were significantly different from the resonant frequencies of the piezo transducer, indicating that the oscillating bubble-liquid system had its own resonant frequencies.

### Resonant frequencies for trapped bubbles in microfluidic system

Operation of bubble-based microfluidic devices at the resonant frequencies of the bubble will optimize efficiency for acoustic-assisted extraction. However, the experimental observations were a result of the combined effects of the piezo transducer and the resonating bubble. In order to characterize the behavior of the microsystem independent of the piezo transducer, a theoretical study is required. Although investigations have been made to theoretically characterize cylindrical bubbles^46^ and untrapped-spherical bubbles^47^,^48^; trapped-semicylindrical-shaped bubbles^45^,^49^ have not been studied yet. In this work, we derive the approximate resonant frequencies of the trapped non-spherical bubbles which arise in liquid-liquid extraction systems.

Bubble oscillations are governed by interactions between acoustic waves, gravity, and capillary waves^47^. Acoustics-based estimates of the resonant frequencies were found to be in the MHz regime whereas our experimental results indicated resonant frequencies in the kHz range^49^,^50^. This is an indication that the bubble frequency response is dominated by capillary forces^49^,^50^. In addition, the effect of the gravitational force is neglected due to a low Bond number (as described in the Supplemental Information).

As the bubble oscillation appears to be governed mainly by capillary wave forces across the interface, we can proceed with an estimate of the bubble resonant frequencies by modeling the bubble as an elementary elastic membrane capable of surface tension but with a negligible intrinsic inertia, and modeling all inertia effects as being due to the fluids on either side of the membrane. This estimation can then be based on the application of conservation of energy for a system consisting of the membrane surrounded by two columns of fluid on either side of this membrane. Clearly, there is fluid motion on the two sides of the interface, but, for the purpose of estimating the resonance frequencies of the bubble, we assume that viscous energy dissipation is a second order effect next to the kinetic energy associated with the harmonic motion of the fluid. That is, in the present calculation we assume that the fluid simply displaces with the membrane. It should be noted that, in the analysis of acoustic streaming, the harmonic motion is generally viewed as the first-order effect, while the streaming is considered a second-order effect^11^. So, referring to Fig. 4a, we write
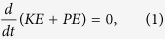
where *KE* and *PE* are the kinetic and potential energies of the system, respectively. As shown in Fig. 4a, the cross-sectional dimensions of the trapped bubble in the *x* and *y* directions are *a* (width of a cavity), and *b* (depth of a cavity), respectively. The shapes of interfaces trapped over a rectangular cross section (*S*) have been modeled previously as^49^:
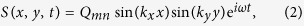
where *ω = 2πf* is the angular frequency, *f* is the frequency, *Q*_*mn*_ is the amplitude, and *k*_*x*_ = *m*π/*a* and *m* are the wave number and mode number, respectively, in the *x* direction. 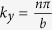 and *n* represent similar quantities in the *y* direction. The variations in the shape of interface *S* are considered to be of the form given in Eq. (2). Adopting an elementary theory of elastic membranes, the pressure difference across the interface is 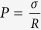, where *σ* is the surface tension and 1/R=*∇ *^2^*S* is the mean curvature of the interface^51^. Thus,
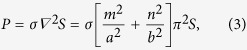
The time rate of change in volume is 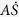, where *A *= *ab* is the cross-sectional area of the side-microtube and the dot above the variable indicates its time derivative. Hence,
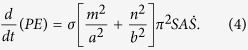
Based on our premise concerning the neglecting of viscous dissipation, the time rate of change of the kinetic energy of our system is the modeled as follows:
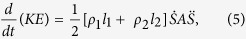
where *ρ*_1_, *ρ*_2_, *l*_1_, *l*_2_ are the densities and the lengths of the respective liquid columns in the side channel. Solving Eqs (1, 4 and 5) for resonant frequencies *f*_*mn*_, we find
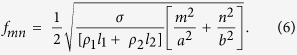
The theoretical resonant frequencies (*f*_*mn*_) for each wave mode (*m, n*) were calculated using the experimental parameters in Table 1 and compared with experimentally determined amplitude peaks as shown in Fig. 4c. We note that each experimental amplitude peak recorded in the frequency range 1-100 kHz matches a calculated *f*_*mn*_ with a combination of *(m, n)*; this suggests that bubble resonance dominates the oscillation behavior. Although experimental identification of the precise resonant mode is difficult using the current characterization method, our theoretical model appears to be sufficient in predicting the amplitude peaks by considering bubble resonances. This model is helpful in optimizing acoustic-activated, trapped bubble systems for not only extraction, but also other fluidic manipulation applications such as mixing and gradient formation.

### Enhancement of mass transfer

In the ALE process, the mass transfer rate is enhanced due to the acoustically-induced streaming. However, due to the randomness of cavitation in the conventional ALE process, it is difficult to quantitatively study the mass transfer enhancement due to the oscillation of single bubbles. This problem can be solved by our on-chip ALE setup, where the trapped bubble is stable and can be actuated with controlled amplitude. A liquid droplet with a defined volume was anchored near the bubble to investigate the mass transfer across the interface.

**Figure 5:**
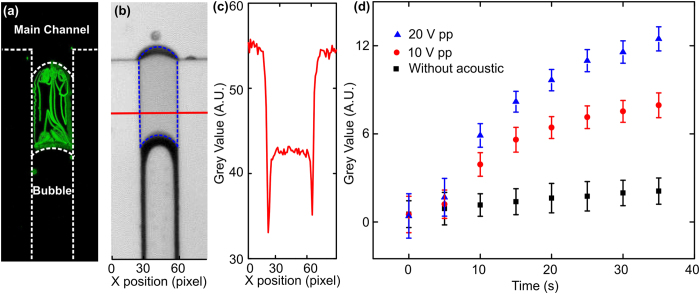
(**a**) A micro-streaming was generated in the sealed liquid droplet when the bubble oscillates. (**b**) Experiments of the mass transfer across the liquid-liquid interface by measuring the grey-scale value of the 1-octanol phase in the sealed droplet, which is enclosed by the blue dotted lines between the bubble and the main channel. (**c**) A plot of average grey-scale values in the sealed droplet along the axis indicated by the red line in (**b**). The difference in grey-scale values between the background (main channel) and the liquid droplet indicates the extent of extraction. (**d**) The changes in the grey-scale value increases with increasing amplitudes of bubble oscillation.

The streaming pattern^52^,^53^,^54^,^55^,^56^,^57^ was visualized by seeding fluorescent beads in the liquid and digitally stacking a time-series of fluorescent images. In the experiment, since polystyrene microparticles could only be dispersed in the aqueous solution, we fabricated an organic, aqueous, gas system. The organic solution (1-octanol) flowed in the main channel, and a small drop of aqueous solution (water) was sealed between the organic phase and the gas bubble. Figure 5a shows that the streamlines were concentrated near the aqueous-gas interface and the organic-aqueous interface, which will facilitate the convective mass transfer across the interface. In addition, the streamlines were spread across the entire aqueous solution, which revealed an evenly distributed solute concentration. This minimizes solute depletion near the interface, thus maintaining a high driving concentration difference for extraction.

Mass transfer enhancement was estimated by measuring the extracted amount of Rhodanmine B from water to 1-octanol (Fig. 5b). Rhodanmine was used as the tracer molecule because its solubility in 1-octanol is much larger than that in water, and it has a strong light absorption property to indicate its concentration. Here, a small drop of 1-octanol was sealed between the aqueous solution (Rhodanmine B dissolved in water, in the main channel) and a gas bubble. In our experiments, the differences in the grey-scale values between the 1-octanol and the background (the averaged grey-scale value from the main channel) were analyzed to determine the concentration of Rhodanmine B (Fig. 5c). Measurements were performed every 5 s after bubble actuation to track the concentration change of the Rhodanmine B in 1-octanol. To determine the influence of the oscillation amplitude on the extraction process, experiments were performed at three different input voltages: 0, 10, and 20 V_pp_. Figure 5d shows the extraction enhancement generated by increasing the input voltages. After 35 s in each experiment, the average grey-scale value in the cavity, which indicates the Rhodanmine B concentration in the 1-octanol, increased about 10 times relative to a non-acoustic-wave-assisted test (a diffusion-driven process). This can be attributed to the higher streaming velocities near the interfaces^52^,^58^,^59^,^60^,^61^,^62^, thereby increasing the rate of molecular transfer from one phase to another. Organic solvent loss was also analyzed by calculating changes in the side-section area of the 1-octanol cavity enclosed by the blue dotted lines in Fig. 5b. Organic solvent loss was minimal for all three test situations: 12% at 20 V_pp_, 14% at 10 V_pp_, and 7% for the non-acoustic-wave-assisted test. A similar low level of organic solvent loss indicated that the extraction extent, rather than organic solvent loss, was the main reason for the increased Rhodanmine B concentration in 1-octanol. Overall, an acoustically-driven, oscillating bubble was shown to significantly increase extraction efficiency with minimum solvent loss.

## Conclusion

In summary, we investigated the mass transfer enhancement and interface oscillations in a liquid-liquid extraction process, with a bubble-based acoustofluidic microsystem. The results show that the extraction efficiency was dependent on the oscillation of the liquid-liquid interface, which was dictated by the characteristic parameters of the bubble. The theoretically calculated resonant frequencies derived from an energy conservation analysis generally matched the frequencies at which the maximum displacement amplitudes were measured over a range of 1–100 kHz. This theoretical analysis can be used in designing acoustic bubble-based systems and selecting the appropriate operating conditions. Rhodanmine B was extracted from water to 1-octanol as a demonstration of the mass transfer enhancement using this method. The mass transfer rate was found to be dependent on the driving voltage. This approach can be potentially used for biomolecule enrichment and for enhancing bio-detection under practical operating conditions. With these functionalities, our device is capable of playing a significant role in investigating many on-chip applications such as biochemical separation, concentration, and bioanalysis.

## Additional Information

**How to cite this article**: Xie, Y. *et al*. Exploring bubble oscillation and mass transfer enhancement in acoustic-assisted liquid-liquid extraction with a microfluidic device. *Sci. Rep*. **5**, 12572; doi: 10.1038/srep12572 (2015).

## Supplementary Material

Supplementary Information

## Figures and Tables

**Table 1:** Parameters to calculate the resonant frequencies of the bubbles.

Parameters	Physical meaning	Values
*σ*	Surface tension of 1-octanol^63^	0.0265 N/m
*ρ*_*1*_	Density of water	1000 kg/m^3^
*l*_*1*_	Length of water in cavity	80 μm
*ρ*_*2*_	Density of 1-octanol	824 kg/m^3^
*l*_*2*_	Length of 1-octanol in cavity	50 μm
*a*	Width of cavity	60 μm
*b*	Depth of cavity	55 μm
