# Supplementary material for: Exploring bubble oscillation and mass transfer enhancement in acoustic-assisted liquid-liquid extraction with a microfluidic device
Source: Sci Rep. 2015 Jul 30;5:12572. doi: 10.1038/srep12572 (PMC4519785; doi:10.1038/srep12572)
Supplement: Supplementary Information [file srep12572-s1.pdf]

Supplementary Information (SI) for *Scientific Reports*

Exploring bubble oscillation and mass transfer enhancements in acoustic-assisted liquid-liquid extraction with a microfluidic device

Yuliang Xie,[a,b] *Chandraprakash Chindam,*[b] *Nitesh Nama,*[b] *Shikuan Yang,*[b] *Mengqian Lu,*[b] *Yanhui Zhao,*[b] *John D. Mai*[c]*, Francesco Costanzo,*[b,d] *and Tony Jun Huang*[*a,b,e]

[a] *Department of Chemical Engineering, The Pennsylvania State University, University Park, Pennsylvania 16802, USA*

[b] *Department of Engineering Science and Mechanics, The Pennsylvania State University, University Park, PA 16802, USA*

[c] Department of Mechanical and Biomedical Engineering, City University of Hong Kong, Tat Chee Avenue, Kowloon, Hong Kong SAR

[d] *Center for Neural Engineering, The Pennsylvania State University, University Park, PA 16802, USA*

[e] *Department of Biomedical Engineering, The Pennsylvania State University, University Park, PA 16802, USA*

* *Corresponding author: Fax: 814-865-9974; Tel: 814-863-4209; E-mail:* junhuang@psu.edu

1. Examples of bubble and liquid-liquid interface oscillations at a single frequency

Figure *S1* described the bubble and liquid-liquid interface oscillation at a single frequency (10.6 kHz). The interface mode was also described in Fig. 4 in the main text, where the aqueous, organic, and gas phases shared one joint point. The oscillation frequency of liquid-liquid interfaces and bubbles were the same as the driven frequency (at 10.6 kHz). The oscillation amplitude for liquid-liquid interface and for bubbles was nearly the same, about 6 μm.

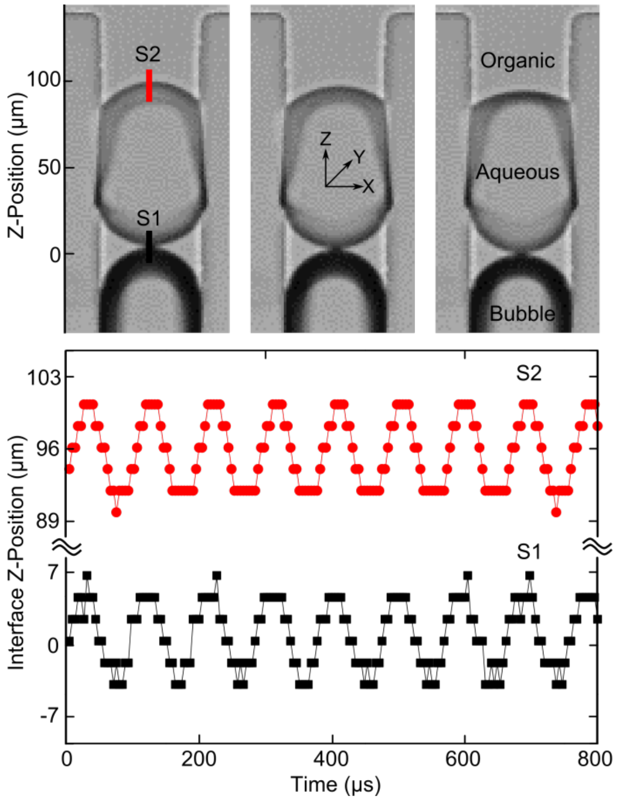

Figure S1 (a) Continuous frames of bubble and interface oscillation at 10.6 kHz, where the organic-aqueous interface (S2) oscillates along the red line, and the aqueous-bubble interface (S1) oscillates along the black line. (b) The displacements of interfaces along the red and black lines were demonstrated with respect to time.

2. Measurements of piezo transducer response at sweep frequencies

The piezo transducer's response to a sweep frequencies signal was studied to determine the resonant frequencies of piezo transducer. To measure the response, two identical piezo transducers were bonded onto one glass slide. One piezo transducer was used to generate the mechanical oscillations, while the other converted the mechanical oscillations into electrical signals. An oscilloscope was connected to the receiving piezo transducer to display the electrical signals. The input electrical signal, with 12 V_{pp}, was swept from frequencies 1 to 100 kHz in 300 ms (red line in Fig. S2a) with a linear increase (blue line) triggered at time point 0 s (black line). shows that The response of the piezo transducer shown in Fig. S2b suggests several resonant peaks within the range 1–100 kHz: 5–6 kHz, 30–35 kHz, 80 kHz, and near 100 kHz. After characterization, an identical piezo transducer was used in the experimental activation of the bubble and liquid-liquid interfaces.

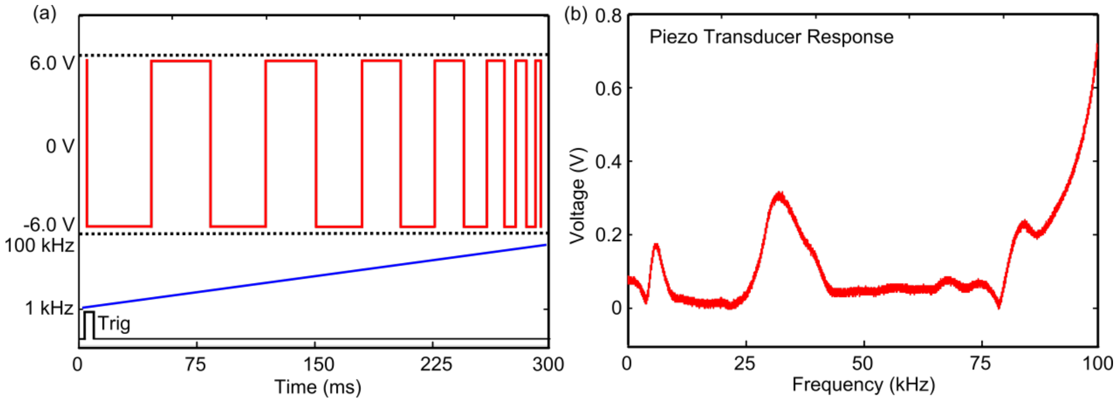

Figure S2 (a) The stimulated signal generated by function generator. (b) The resonant frequency of the piezo transducer under the stimulated wave in Fig. S2a.

3. Calculation of Bond number in the oscillating system

The Bond number of the oscillating system can be calculated as:

$$Bo = \frac{\rho g L^2}{\gamma} \tag{S1}$$

where *Bo* is the dimensionless Bond number; ρ is the density (824 kg/m^3 for 1-octanol); g is the gravity (9.8 m/s^2); L is characteristic length (the width of the side wall channel, 60 μm); and γ is the surface tension (0.0265 N/m for 1-octanol). The calculated Bond number was 0.001, significantly less than 1. When the liquid was water, the Bond number is also significantly less than 1. These results indicate that the effect of gravity can be neglected when surface tension is taken into consideration.
